# The Effects of Job Insecurity on Psychological Well-Being and Work Engagement: Testing a Moderated Mediation Model

**DOI:** 10.3390/bs15070979

**Published:** 2025-07-19

**Authors:** Maria Leonor Pires

**Affiliations:** ESTSetúbal, Instituto Politécnico de Setúbal, 2910-761 Setúbal, Portugal; leonor.pires@estsetubal.ips.pt

**Keywords:** job insecurity, psychological well-being, work engagement, work self-efficacy, supervisor support

## Abstract

In recent decades, there have been significant changes in employment relationships, leading to more precarious forms of employment and heightened perceptions of job insecurity. The purpose of this study was to test the effects of job insecurity on perceived psychological well-being and work engagement, with self-efficacy at work acting as a mediator and perceived supervisor support acting as a moderator. This study analyzed a sample of 938 individuals from Portugal who participated in the 2015 European Working Conditions Survey (EWCS). The results show that job insecurity has a negative impact on both psychological well-being and work engagement. Furthermore, the study found that self-efficacy at work acts as a mediator in the relationship between job insecurity, psychological well-being, and work engagement, with a buffering effect. Our results also show evidence of a moderation effect of perceived supervisor support, which works in two ways: perceived supervisor support amplifies the positive effect of self-efficacy at work on psychological well-being, as well as the indirect negative effect of job insecurity. However, this moderation effect was not observed in the relationship with work engagement. These results emphasize the significance of job insecurity regarding individual mental health and job-related attitudes, taking into consideration individual and organizational resources.

## 1. Introduction

When an individual’s family income depends on them having a paid job, the prospect of unemployment can be very worrying, to say the least. For today’s employees, this scenario can be very real and may repeat itself throughout their working lives, since employment relationships have undergone major changes in recent decades. The shift in the traditional job model, driven by economic crises, companies’ quests to achieve a competitive edge, and technological advancements, has resulted in heightened job insecurity, even among those who are currently employed (Chirumbolo & Hellgren, 2003). These transformations carry significant implications for individuals and organizations alike. Research has shown that job insecurity can significantly impact physical and mental health. Studies have reported physical troubles (back problems, muscular pain, headaches, eyestrain, and stomachache); depression or anxiety; overall fatigue; insomnia (Caroli & Godard, 2016); and lower mental well-being (De Witte et al., 2015).

Job insecurity also has a negative impact on attitudes and behaviors at the organizational and work-related levels. These include a decline in organizational commitment, distrust in management, resistance against organizational change, fewer organizational citizenship behaviors, and a stronger intention to leave (De Witte, 2005), as well as reduced job satisfaction, absenteeism (De Witte et al., 2015), more negative attitudes toward work, and lower performance, creativity, and adaptability (Shoss, 2017).

The Portuguese sovereign debt crisis that occurred between 2011 and 2014 led the country to request financial assistance from the European Commission, the European Central Bank, and the International Monetary Fund, known as the Troika intervention, which had important consequences for the Portuguese labor market. Alterations in legislation (Cardoso & Branco, 2018) led to important changes in employment protection, unemployment security, and collective bargaining. There was a reduction in job security and protection for the unemployed and a loss of negotiating power for workers’ representatives vis-à-vis employers. Part of the reforms concerning the labor market were aimed at lowering the costs of dismissing regular and temporary workers for employers, including weakening protection against unfair dismissal and extending the duration of fixed-term employment contracts. Unemployment income was reduced, as was the duration of access to this income, both for permanent and temporary workers. The unemployment rate rose from 12.8% in 2011 to a peak of 16.5% in 2013, with a rate of 12.5% in 2015. Considering that the economic context has an important role in shaping workers’ job insecurity, the 2015 European Working Conditions Survey (EWCS) seems appropriate for exploring workers’ perceptions of job insecurity and its consequences.

This study employed the conservation of resources (COR) theory as its theoretical framework; a moderated mediation model was developed to examine self-efficacy at work as a mediator between job insecurity, the predictor, and the two criterion variables, psychological well-being and work engagement, with perceived supervisor support acting as a moderator of the mediation relationships. This study contributes to the existing literature by clarifying how and why job insecurity affects individual and organizational outcomes, thus broadening our knowledge of the mechanism through which individual and organizational resources impact these outcomes. We found that job insecurity adversely affects self-efficacy, psychological well-being, and work engagement. Self-efficacy serves as a mediator, mitigating the impact of job insecurity. Increased perceived supervisor support enhances self-efficacy and psychological well-being, although the adverse effects of job insecurity remain significant. Furthermore, self-efficacy offers protective benefits for work engagement in the context of job insecurity. However, this research only partially confirmed moderated mediation effects.

## 2. Literature Review and Hypotheses

### 2.1. Job Insecurity

The concept of job insecurity is defined as a perception of impotence in maintaining an employment relationship in a situation of threat to the current job—a quantitative dimension (Greenhalgh & Rosenblatt, 1984)—or the perception of a threat to one’s job conditions, autonomy, or resources—a qualitative dimension (Hellgren et al., 1999; Shoss, 2017). This uncertainty is a characteristic of the working environment as it is perceived by an individual (Greenhalgh & Rosenblatt, 1984). It can vary according to the severity of the threat to the job itself or its conditions (Hellgren et al., 1999).

The sources of perceived job insecurity can be identified at three levels (De Witte, 2005): the macrolevel, the level of individual characteristics, and the level of personality traits. At the macrolevel, perceived job insecurity can be influenced by either national, regional, or organizational variables (such as economic conditions, the unemployment rate, or organizational changes); at the individual level, it is related to the job position an individual occupies and their demographics; and, finally, personality traits include the person’s locus of control or affectivity and dispositions (Bosman et al., 2005).

Several mechanisms by which job insecurity influences individual attitudes, behaviors, and work-related outcomes have been described in the literature. From a social exchange perspective, it involves the perception of a psychological contract breach (De Witte, 2005; Shoss, 2017), since security is one of the benefits that employees expect from their contractual relationship with a company. There can also be a sentiment of injustice, since a reduction in the benefits or rewards associated with a job is likely to create the perception of an imbalance between duties and rewards.

Our research adopted the conservation of resources (COR) theory as a theoretical framework. This theory was first presented as a stress model, according to which “people strive to retain, protect and build resources and what is threatening to them is the potential or actual loss of these valued resources” (Hobfoll, 1989).

Such resources include objects, valued conditions, personal characteristics, and energies that are valued per se or because they lead to the obtention or protection of valued resources (Hobfoll, 1989, 2001, 2002). Stress occurs when individuals experience a loss of or threat to their resources. There are two crucial aspects of this theory: first, there is the principle that resource loss has a greater impact on an individual than resource gain does, thus increasing a stress response; second, individuals tend to invest in resources that can act as a buffer to resource loss and, therefore, to stressors (Hobfoll, 2001).

In modern economies, a job is a source of valued resources, providing funds, means of subsistence, social status, social integration, and the acquisition as well as use of skills, to name a few aspects, leading to better well-being. Job insecurity represents a threat to these valued resources, thereby inducing stress. Individuals must also deal with unpredictability—not knowing what will happen makes it difficult to act adequately—and uncontrollability—or the powerlessness to deal with a threat—both of which have a negative effect on well-being; additionally, feelings of deprivation can emerge (De Witte, 2005). The consequences at the individual level are negative; workers may experience higher levels of anxiety, depression, absenteeism, and turnover intentions; lower performance, satisfaction, and engagement (Jiang & Lavaysse, 2018; Sverke & Hellgren, 2002); worse physical and mental health; psychological stress; burnout; lower self-esteem; and worse family relationships (Chirumbolo & Areni, 2010).

At the organizational level, job insecurity is predicted to weaken job attitudes and contributions to one’s organization (Shoss, 2017), with less involvement and motivation, lower performance (De Witte, 2005), greater resistance to organization changes (Greenhalgh & Rosenblatt, 1984), less commitment to and confidence in the organization, and lower job satisfaction (Ashford et al., 1989). A meta-analysis (Sverke et al., 2019) found that job insecurity was significantly associated with decreased employee performance in both cross-sectional and longitudinal studies, with most of the findings confirming negative associations with several types of employee performance (such as task performance, contextual performance, and safety performance).

Employees can also engage in behaviors directed at job preservation and active coping strategies (Shoss, 2017). Employees who still hope to avoid dismissal may devote extra effort toward behaviors that will be noticed and valued (e.g., enhanced task performance and organizational citizenship behavior), attempt to portray themselves as an ideal employee, voluntarily intensify work efforts (Selenko et al., 2013), work excessively (Finstad et al., 2024), and refrain from behaviors that would draw negative attention. They may also engage in presenteeism, or collective action, by joining unions (Shoss, 2017). In the attempt to cope with the prospect of job loss, individuals can also pursue job opportunities or invest in education or training; however, these initiatives may lead to the depletion of already-diminishing resources, which intensifies stress when combined with the prospect of job loss (Halbesleben & Buckley, 2004).

At the same time, there are other important resources that one may possess or cultivate to maintain a reserve of resources, such as self-efficacy, social support, self-esteem, and optimism (Hobfoll, 2001, 2002), thus avoiding or counteracting resource loss (Halbesleben et al., 2014).

Research has pointed out various possible intervening variables in the relationships between job insecurity and work-related outcomes. At the individual level, personality differences, emotional intelligence, a locus of control, and the need for security, among others, can shape an individual’s responses to the prospect of job loss; at the organizational level, the mediators that have been studied include social support, work control, participation in decisions, fair treatment, and organizational justice (Chirumbolo & Areni, 2010).

In this study, we examined self-efficacy at work as a mediator between job insecurity and two outcomes, psychological well-being and work engagement, while testing perceived social support from a supervisor as the moderator.

Job insecurity starts a loss spiral, since it threatens a fundamental resource: job stability. According to the COR theory, when employees face a threat to their resources, they take a defensive stance and engage in conservation behaviors to prevent additional resource depletion (König et al., 2010; Salanova et al., 2010), such as reducing efforts in task performance and organizational citizenship behaviors (OCBs), with the intent to preserve their remaining resources, such as time and energy (König et al., 2010). However, this withdrawal behavior may exacerbate strain, since diminished performance can lead to poorer evaluations, thus reinforcing perceptions of insecurity and continuing the loss cycle (Debus & Unger, 2017).

The way to mitigate resource loss spirals is through resource accumulation. For this purpose, self-efficacy can act as a personal resource by promoting coping strategies that allow individuals to adapt better. Individuals with high self-efficacy are more likely to interpret job insecurity as a challenge rather than a threat, investing resources proactively to secure alternative outcomes (König et al., 2010; Salanova et al., 2010). Being proactive interrupts the loss spiral, enables performance, and reduces strain (König et al., 2010).

A contextual resource that reinforces individual resources is supervisor support. Supervisors can provide resources such as information on changes, thus heightening the perception of control, or reassurance, which reduces uncertainty (Debus & Unger, 2017; Salanova et al., 2010). If employees perceive insecurity as manageable, they will be encouraged to continue to invest resources in their roles (König et al., 2010). This aligns with the concept of “resource caravans,” where contextual resources such as supervisor support aggregate with personal resources to foster resilience (Salanova et al., 2010).

The interaction between self-efficacy and supervisor support creates positive feedback loops, leading to resource gains. Workers with high self-efficacy effectively use support from supervisors, seeking feedback to deal with insecurity (Salanova et al., 2010). Supervisors who offer support increase employees’ self-efficacy by recognizing achievements and providing growth opportunities. This synergy generates a spiral of gains, improving adaptability to job insecurity (Debus & Unger, 2017; Salanova et al., 2010).

### 2.2. Self-Efficacy at Work

Self-efficacy refers to the “belief in one’s capability to organize and execute the courses of action required to produce given attainments” (Bandura, 1997). Perceptions of self-efficacy can affect stress levels and the physical health of workers, so that individuals with lower levels of self-efficacy are more prone to displaying higher levels of anxiety and more health problems (Bandura, 1997). On the contrary, higher levels of self-efficacy positively influence stress, job satisfaction, and general well-being (Grau et al., 2001; Salanova et al., 2006).

In the literature (A. B. Bakker & Demerouti, 2008), the role of personal resources (including, optimism and self-efficacy) or positive self-evaluations (which foster resilience and positive self-regard), as well as self-concordance of goals, and their association with positive outcomes (such as motivation, performance, and job satisfaction) have been emphasized. There is also evidence that self-efficacy is a predictor of work engagement (Bresó et al., 2011; Xanthopoulou et al., 2007, 2009).

However, organizational conditions, such as a heavy workload, poor career expectations, and an unsatisfying work–life balance, can weaken self-efficacy (A. B. Bakker & Demerouti, 2008). We believe that the prospect of losing one’s job can induce all three of these conditions: an employee can accept a heavier workload to try to avoid being fired; actually leaving a job will cause uncertainty regarding career development; and feelings of imbalance between one’s work and personal life can emerge or be accentuated.

Research by Yao et al. (2021) found a negative correlation between job insecurity and self-efficacy; moreover, this particular study revealed a significant negative effect of job insecurity on employees’ proactive behavior, with self-efficacy significantly moderating the relationship between job insecurity and the assessment of hindrance, as lower levels of self-efficacy increase the likelihood of job insecurity being perceived as a hindrance. Self-efficacy also moderated the mediating role of obstacle evaluation between job insecurity and proactive behavior, with a higher mediation effect at lower levels of self-efficacy.

However, this relationship may not always be straightforward, as suggested by Adewale et al. (2019), who failed to find a significant direct relationship between self-efficacy and job insecurity, while admitting that a non-linear relationship could exist between the two constructs.

In this research, we used the concept of self-efficacy at work. Professional self-efficacy at work is defined as “feelings of competence and successful achievement in one’s work” (Greenglass et al., 2003). This concept corresponds to one of the burnout dimensions proposed in a previous study (Leiter & Schaufeli, 1996), with the others being emotional exhaustion and cynicism. When burnout occurs, feelings of exhaustion and cynicism are high and professional self-efficacy is low. The results of research conducted by Greenglass et al. (2003) with a sample of nurses demonstrated that the workload acted as a stressor diminishing the feeling of professional efficacy, but at the same time, professional efficacy had a negative effect on depression, meaning that nurses with higher levels of professional self-efficacy exhibited lower depression scores. The results from another study (Guarnaccia et al., 2018), measuring the effects of job insecurity on job satisfaction, work engagement, and general health through the mediation of occupational self-efficacy, evidenced the negative consequences of job insecurity on all three outcomes, while a partial mediation role of occupational self-efficacy was also confirmed.

### 2.3. Psychological Well-Being

Well-being can be defined as “a subjective feeling state in which positive feelings predominate”, implying a positive self-appraisal, and it can be assessed in different domains of health, including the emotional domain (McDowell, 2010; The Whoqol Group, 1998), which is included in the World Health Organization (WHO)’s general definition of health as “a state of complete physical, social and mental well-being, and not merely the absence of disease or infirmity” (World Health Organization, 1952).

Over the years, research has found that higher levels of perceived job insecurity led to worse physical and mental health, psychological stress, lower self-esteem, and worse family relations (Chirumbolo & Areni, 2010). A recent study (Kromydas et al., 2021) found evidence that becoming unemployed was strongly associated with common mental disorders, with men being more sensitive to employment transitions.

A study using cross-sectional data from 22 European countries (Caroli & Godard, 2016) confirmed the health-damaging effects of job insecurity on physical health, such as suffering from headaches, eyestrain, and skin problems.

There is also evidence from an Italian study (Pirani & Salvini, 2015), where a negative association between temporary employment and general health was identified. The study also found that the more prolonged this form of employment is, the more damaging it is for an individual’s health; additionally, women seem to be more negatively affected than men by temporary employment.

In terms of mental health, (Watson & Osberg, 2018) an existing study found that job insecurity is associated with an increase in psychological distress, whereas the results regarding the effect of unemployment were not as conclusive, which highlights the importance of perceived threat and uncertainty in the unemployment situation in itself. A meta-analysis study (De Witte et al., 2016) based on 57 published studies showed that job insecurity was significantly associated with anxiety, after controlling for baseline mental health and psychosomatic complaints, as well as flu-like illness, gastroenteritis, and health complaints over time.

A study by Yoo (2023) investigated job stressors in the hotel industry and their impact on workers’ psychological well-being. Job insecurity significantly increased stress, which acted as a mediator between insecurity and psychological well-being, indicating that job insecurity indirectly affects well-being by exacerbating work stress.

Concerning employment status, a longitudinal study (Hammarstrom et al., 2011) found that perceived job insecurity had adverse effects on self-rated suboptimal health, psychological distress, and depressive symptoms among both permanent and temporary employees, with the effect of job insecurity on health being the same in both groups.

The study on job insecurity among precarious Malaysian workers by Abdul Jalil et al. (2023) found that insecurity was negatively associated with both a work–life balance and psychological well-being. The research also highlighted that work–life balance significantly mediated the relationship between job insecurity and psychological well-being, underscoring the importance of a work–life balance in mitigating the adverse effects of job insecurity on psychological well-being.

There is also evidence that job insecurity correlates negatively with well-being at work (Castro-Castañeda et al., 2023). Workers with stronger perceptions of job insecurity feel that they lack the technical, social, and labor resources that are needed for their performance and well-being; they view their workplace as uncomfortable, unhealthy, and unsafe.

Furthermore, a study on the effects of job stress on burnout and turnover (Üngüren et al., 2024) concluded that high job security can buffer the impact of job stress on burnout, while high job insecurity combined with job stress increases the risk of burnout.

### 2.4. Work Engagement

Work engagement is a positive motivational state that is characterized by elements such as enthusiasm, dedication, and absorption (A. B. Bakker et al., 2014). It has been associated with positive work-related attitudes, such as greater job satisfaction and commitment, organizational citizenship behaviors, lower turnover, and higher job performance (Rich et al., 2010). The antecedents encompass organizational and work-related attributes. These include perceived social support provided by colleagues and supervisors, the diversity and significance of tasks, autonomy, and feedback, as well as appreciation and the organizational climate (A. B. Bakker & Demerouti, 2008).

Job insecurity represents a significant threat to an employee’s resources, particularly those related to career stability and future prospects (De Witte et al., 2015). When faced with such threats, individuals tend to adopt a defensive posture, focusing on resource conservation rather than resource investment (Hobfoll et al., 2018). This defensive stance can manifest as reduced work engagement, as employees divert their cognitive and emotional resources towards maintaining employment rather than fully immersing themselves in their work roles (Kinnunen et al., 2011). In addition, in line with the COR theory, employees experiencing job insecurity may adopt resource investment strategies that prioritize low-risk, predictable outcomes over potentially beneficial but uncertain work engagement behaviors (Hobfoll et al., 2018). This risk-averse approach can result in reduced proactive work behaviors and diminished overall engagement (Probst et al., 2016).

Negative relationships between work engagement, burnout, and stress at work have been reported (Maslach et al., 2001; Schaufeli & Salanova, 2008). Considering that job insecurity can be perceived as a stressor, with the potential to trigger negative emotions, it can also have the effect of weakening work engagement (Guarnaccia et al., 2018; H. Wang et al., 2015) and, consequently, be detrimental to engagement-related positive attitudes, leading to higher levels of intention to quit (Mer et al., 2023).

### 2.5. Perceived Supervisor Support

Several resources have been identified that can enhance stress resistance, including individual resources, such as self-efficacy, and broader organizational support, described as a “broadband resource” (Hobfoll, 2001). Organizational support is further categorized (Demerouti et al., 2001) as a component of job resources, which are elements of a job—encompassing physical, psychological, social, or organizational aspects—that can mitigate job demands and their associated costs. In a previous study (A. B. Bakker & Demerouti, 2008), the motivational role of resources such as social support was highlighted, aiding to achieve work goals and increasing employees’ willingness to exert effort, as well as utilize their abilities, thereby fostering work engagement.

Perceived organizational support can be defined (Rhoades & Eisenberger, 2002) as employees’ general beliefs about the extent to which their organizations value their contributions and care about their well-being. As representatives of an organization, supervisors can signal organizational support, leading employees to develop similar perceptions about organizations.

Empirical research has consistently demonstrated the positive impact of organizational support on individuals’ perceptions, attitudes, and behaviors (Rhoades & Eisenberger, 2002). In addition, supervisor support has been negatively correlated with exhaustion, while coworker support has been positively correlated with self-efficacy.

Nelson & Smith (Nelson & Smith, 2023) found that support in the workplace directly enhances psychological well-being and reduces distress. However, there was no direct correlation between work support and overall physical health, while job satisfaction played a significant mediating role in the relationship between work support and both positive well-being and general physical health.

Perceived supervisor support can exert a positive influence on work engagement when supervisors provide emotional and instrumental resources to employees, thereby enhancing their capacity to fully engage in their work (A. B. Bakker & Demerouti, 2017; A. Bakker et al., 2003). Supervisor support can also facilitate employees’ conservation of resources, either by mitigating job demands or furnishing resources to meet those demands, thus enabling employees to engage in their tasks (Airila et al., 2014).

These findings collectively suggest that support has a positive influence on work engagement (Leiter & Maslach, 2003). Other studies (Schaufeli & Bakker, 2004) also observed a positive relationship between supervisory coaching and work engagement.

Longitudinal studies (Mauno et al., 2007; Schaufeli et al., 2009) confirmed the positive associations between job resources and work engagement. Specifically, it was found (Schaufeli et al., 2009) that an increase in job resources predicts higher levels of work engagement, while a decrease predicts higher levels of burnout.

It was further demonstrated (Caesens & Stinglhamber, 2014) that there was a positive relationship between perceived organizational support and work engagement, with this relationship being partially mediated by self-efficacy. Additionally (Vander Elst et al., 2016), it was found that job resources are associated with higher work engagement and lower burnout among healthcare employees. The study also revealed that social support moderates the relationship between workload and burnout, with the association being insignificant for individuals with high levels of social support.

In addition, while testing the impact of perceived social support on job satisfaction through the mediation of work engagement on a sample of teachers, Z. Wang et al. (2024) found that perceived social support was a predictor of work engagement, and that the relation with job satisfaction was indirect, mediated by work engagement.

Moreover, perceived supervisor support contributes to enhanced well-being by serving as a buffer against job stressors, including job insecurity. This support mechanism provides resources that aid employees in managing work-related challenges, consequently diminishing psychological distress and promoting overall well-being. Beyond its stress-buffering effects, supervisor support also bolsters self-efficacy beliefs, which are linked to superior mental health outcomes and increased resilience when confronted with job insecurity (Chen et al., 2015).

A longitudinal study (Hamzah & Nordin, 2022) revealed that perceived supervisor support significantly predicted work engagement among university staff through the mediating role of job-related affective well-being. Similarly (Nabawanuka & Ekmekcioglu, 2022), research on millennials demonstrated that perceived supervisor support enhanced employees’ work–life balance, which subsequently improved their well-being by reducing the roles of conflict and emotional exhaustion. Conversely, a cross-sectional study of Swiss employees highlighted that a lack of supervisor support tripled the risk of burnout and job dissatisfaction (Hämmig, 2017).

### 2.6. Research Model and Hypotheses

Following previous findings in the literature, we tested a model examining the relationships between job insecurity (predictor), psychological well-being, and work engagement (criterion variables), with self-efficacy acting as a mediator. Additionally, we examined the moderating role of perceived supervisor support in the relationships between job insecurity and both psychological well-being and work engagement through self-efficacy at work.

**Figure 1:**
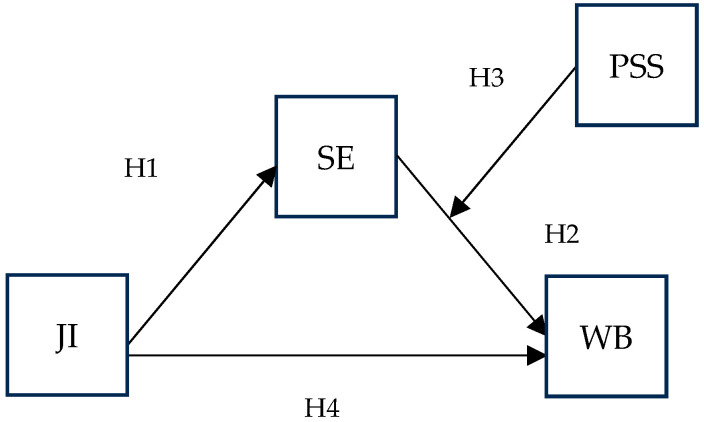
Research model for well-being. Acronyms: JI—job insecurity; SE—self-efficacy at work; PSS—perceived supervisor support; WB—psychological well-being.

As we tested the effects of the predictor on the two criterion variables separately, the hypotheses concerning psychological well-being (Figure 1) were as follows:

**H1.**  *Job insecurity negatively affects self-efficacy at work*.

**H2.**  *Self-efficacy at work positively affects psychological well-being*.

**H3.**  *Perceived supervisor support moderates the effect of self-efficacy on psychological well-being; the positive effect is stronger at higher levels of supervisor support*.

**H4.**  *Perceived supervisor support moderates the indirect effect of job insecurity on psychological well-being through self-efficacy, so that the indirect effect is weaker when perceived supervisor support is high*.

**Figure 2:**
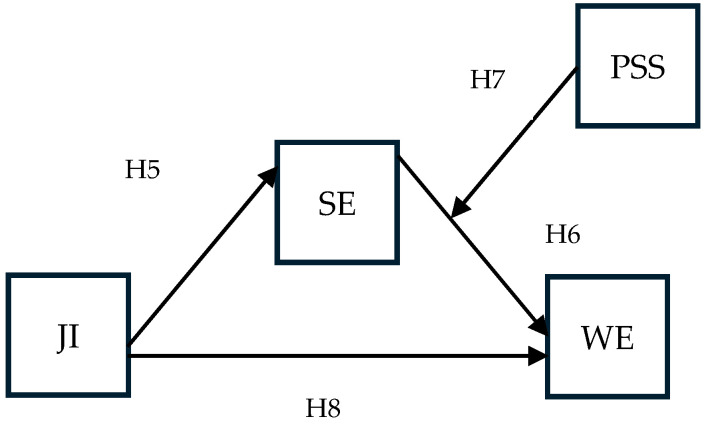
Research model for work engagement. Acronyms: JI—job insecurity; SE—self-efficacy at work; PSS—perceived supervisor support; WE—work engagement.

The hypotheses relating to work engagement (Figure 2) are as follows:

**H5.**  *Job insecurity negatively affects self-efficacy at work*.

**H6.**  *Self-efficacy at work positively affects work engagement*.

**H7.**  *Perceived supervisor support moderates the effect of self-efficacy on work engagement; the positive effect is stronger at higher levels of supervisor support*.

**H8.**  *Perceived supervisor support moderates the indirect effect of job insecurity on work engagement through self-efficacy, so that the indirect effect is weaker when perceived supervisor support is high*.

## 3. Materials and Methods

### 3.1. Sample and Procedures

The data for this study were taken from the Portuguese sample of the 6th European Working Conditions Survey (EWCS), conducted in 2015 by Eurofound. Data were collected through face-to-face interviews with a standardized questionnaire, following a stratified random sampling design to ensure national representativeness. The Portuguese version was developed through independent parallel translations, expert review, and cognitive tests to check clarity and comprehension. The EWCS scales have been widely used and evaluated in previous research, generally showing acceptable to good reliability in national samples, including Portugal.

The current investigation utilized a sample of 938 Portuguese individuals. The average age of the participants was 41.4 years, with 43.4% of the sample being males. Most of the participants (74.4%) were employed in the private sector, and 63.9% held permanent job contracts; the average tenure was 14.5 years. The respondents’ most prevalent level of education was upper secondary education (63.2%).

In the survey, participants were asked to evaluate their perceptions of job insecurity, psychological well-being, work engagement, and self-efficacy at work using Likert-type scales ranging from 1 to 5. Where necessary, the items were recoded to ensure that higher scale values consistently represented a greater degree of agreement.

### 3.2. Measures

The scales employed in our study were constructed using items directly derived from the EWCS 2015 questionnaire. These items were selected based on their theoretical relevance to the constructs under investigation and guided by prior research that has used the EWCS in similar contexts.

Job insecurity: Job insecurity was measured using one item, “I may lose my job in the next 6 months”, with a Likert-type scale of 1 (strongly disagree) to 5 (strongly agree).

Self-efficacy at work: Self-efficacy at work was measured with one item, “In my opinion, I am good at my job”, with a Likert-type scale of 1 (strongly disagree) to 5 (strongly agree).

Perceived supervisor support: Perceived supervisor support was measured with the following six items: “Your immediate boss respects you as a person”; “Your immediate boss gives you praise and recognition when you do a good job”; “Your immediate boss is successful in getting people to work together”; “Your immediate boss is helpful in getting the job done”; “Your immediate boss provides useful feedback on your work”; and “Your immediate boss encourages and supports your development”. Each question was measured with a Likert-type scale of 1 (“strongly agree”) to 5 (“strongly disagree”). The assessment of the one-dimensionality of this measure was carried out using exploratory factor analysis (EFA), which showed the presence of item correlation (Bartlett’s test showed a significance level of 0.000, and the KMO was 0.912); the total percentage of variance explained by this single factor was 71%. This measure showed good levels of internal reliability, with a Cronbach’s alpha = 0.91.

Work engagement: Work engagement was measured with three items, all using a Likert-type scale ranging from 1 (“strongly disagree”) to 5 (“strongly agree”): “Time flies when I am working”, “At my work I feel full of energy”, and “I am enthusiastic about my job”. The assessment of the one-dimensionality of this measure was carried out through exploratory factor analysis (EFA), and the results indicated the presence of item correlation (Bartlett’s test showed a significance level of 0.000, and the KMO was 0.67, which were deemed acceptable). The total percentage of variance explained by this single factor was 69.3%. This measure demonstrated adequate levels of internal consistency, with a Cronbach’s alpha coefficient of 0.78.

Well-being: Psychological well-being was evaluated with the World Health Organization Five Well-Being Index (WHO-5), which also comprised a Likert-type scale ranging from 1 (“strongly disagree”) to 5 (“strongly agree”). One of the sample items was “I have felt cheerful and in good spirits”. The assessment of the one-dimensionality of this measure was carried out through exploratory factor analysis (EFA), and the results indicated the presence of item correlation (Bartlett’s test showed a significance of 0.000, and the KMO was 0.89, which was considered good). The total percentage of variance explained by this single factor was 72.4%. This measure demonstrated adequate levels of internal consistency, with a Cronbach’s alpha coefficient of 0.90.

For control variables, we used age, sex, type of job contract (temporary/permanent), and tenure.

Since all the variables in this study were sourced from the same set of respondents, we acknowledge the potential for common method variance. To mitigate this concern, we conducted Harman’s single-factor test (Harman, 1967), which revealed 9 factors, with the primary factor accounting for only 23% of the total variance.

In addition to Harman’s test, the marker variable technique was employed, as recommended by Lindell and Whitney (2001). A theoretically unrelated marker variable, “involvement in voluntary or charitable activity”—with a Likert scale of 1 to 5, as for our variables), was used to further assess the extent to which common method variance may have influenced the results. Following Lindell and Whitney’s (2001) suggested approach, partial correlations were computed between the main study constructs while controlling for the marker variable. The results indicated that the correlations among the principal variables of interest remained significant and substantively unchanged after controlling for the marker variable. Despite the possibility of common method bias, we believe that it did not significantly influence the study’s results.

### 3.3. Data Analysis

Statistical analysis for this study was performed using SPSS 29.0 software. Descriptive statistics, correlations, and hierarchical regression were first used to test the relationships between job insecurity, self-efficacy, well-being, work engagement, and supervisor support. Next, we used the PROCESS macro developed by Hayes (2017) to estimate the hypothesized moderated mediation model of relationships.

## 4. Results

### 4.1. Descriptive Statistics and Correlation Analysis

The results of the descriptive statistics and correlation analysis, with means, standard deviations, and Cronbach’s alphas, are reported in Table 1. As the results show, job insecurity is negatively correlated with self-efficacy at work (r = −0.144; *p* < 0.01), work engagement (r = −0.208; *p* < 0.01), and psychological well-being (r = −0.240; *p* < 0,01). The results also show that self-efficacy at work is positively correlated with perceived supervisor support (r = 0.195; *p* < 0.01), work engagement (r = 0.560; *p* < 0.01), and well-being (r = 0.302; *p* < 0.01).

### 4.2. Hypothesis Testing

The process of hypothesis testing was carried out in several steps: a first step with hierarchical regression analyses, and a second one using PROCESS, a macro that is available for the SPSS software (Hayes, 2017). Before conducting linear regression analyses, the assumptions underlying the model, specifically the normality of the data, homogeneity, and error independence, were systematically evaluated to ensure the appropriateness and robustness of the results.

The outcomes of these analyses are presented in the subsequent tables.

As presented in Table 2, we found that age has a negative effect on psychological well-being perceptions, but this effect is reduced after other explanatory variables have been entered into the model. As for the type of job contract, it is evident that having a permanent contract has a positive effect on psychological well-being; however, when other explanatory variables have been accounted for, it loses its significance.

Regarding the predictor variable, we found that job insecurity has a negative direct effect on psychological well-being in model 2 (β = −0.230; *p* < 0.001), but this effect decreases in models 3 and 4 after self-efficacy at work and perceived supervisor support have been entered into the models. Additionally, self-efficacy has a positive direct effect on well-being (model 3: β = 0.282, *p* < 0.001; model 4: β = 0.239, *p* < 0.001), as well as supervisor support (β = 0.335; *p* < 0.001). This justifies further assessment of the existence of mediation and moderation effects.

For work engagement, the results are similar. Regarding the control variables, having a permanent job contract has a positive effect on work engagement (model 1: β = 0.138, *p* < 0.001; model 2: β = 0.104, *p* < 0.001) that becomes non-significant in later models. The direct effect of job insecurity on work engagement is negative (β = −0.202; *p* < 0.001), which is reduced but remains significant in subsequent models (model 3: β = −0.125, *p* < 0.001; model 4: β = −0.062, *p* < 0.01); this effect, in conjunction with the positive direct effects of self-efficacy at work (model 3: β = 0.540, *p* < 0.001; model 4: β = 0.491, *p* < 0.001) and perceived supervisor support (β = −0.347; *p* < 0.01) on work engagement, supports further evaluation of the presence of mediation and moderation effects.

First, to test the hypothesized direct and indirect effects, we used PROCESS macro model 4 (Hayes, 2017). The effects of job insecurity on psychological well-being and work engagement were analyzed separately.

#### 4.2.1. Hypothesis Testing for Well-Being

The results (see Table 3) support H1, with a significant negative direct effect of job insecurity on self-efficacy (B = −0.099; *p* < 0.001). H2 was also supported, with self-efficacy exerting a significant positive effect on psychological well-being (B = 0.290; *p* < 0.001). The mediation hypothesis (H3) was supported: job insecurity had a significant direct effect on well-being (B = −0.161; *p* < 0.001) and a significant indirect effect through self-efficacy (B = −0.037).

To test for moderated mediation as predicted in H4, we used PROCESS macro model 14. The interaction between self-efficacy and perceived supervisor support was significant (B = 0.120; *p* < 0.001), indicating that the positive effect of self-efficacy on well-being is stronger when perceived supervisor support is higher. Regarding the conditional indirect effect, job insecurity’s indirect effect on psychological well-being was more pronounced at higher levels of supervisor support, thus partially supporting H4.

**Figure 3:**
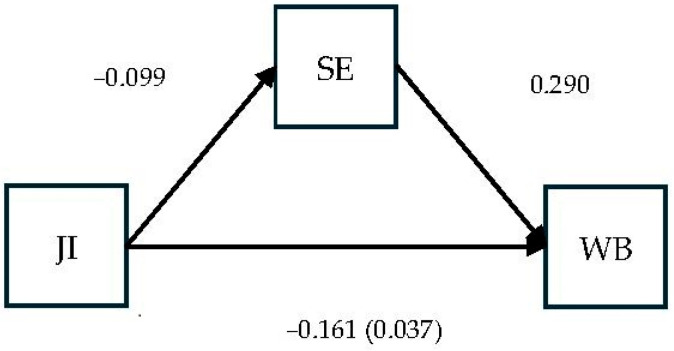
Mediation results for psychological well-being (indirect effect in brackets).

**Figure 4:**
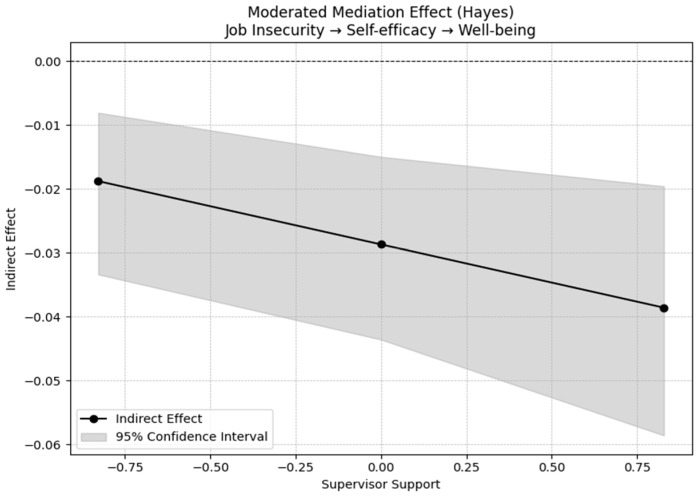
Moderated mediation effect of job insecurity on psychological well-being through self-efficacy at different levels of supervisor support.

Figure 3 and Figure 4 below provide a visual representation of the mediation results and offer additional support for the interpretation of the moderated mediation results.

#### 4.2.2. Hypothesis Testing for Work Engagement

The same analytic procedures were applied for work engagement. As shown in Table 4, H5 was supported: job insecurity negatively predicted self-efficacy at work (B = −0.099; *p* < 0.001). H6 was also supported, as self-efficacy positively predicted work engagement (B = 0.470; *p* < 0.001). The direct (B = −0.085; *p* < 0.001) and indirect (B = −0.050; *p* < 0.001) effects of job insecurity on work engagement were both significant, supporting H7.

**Figure 5:**
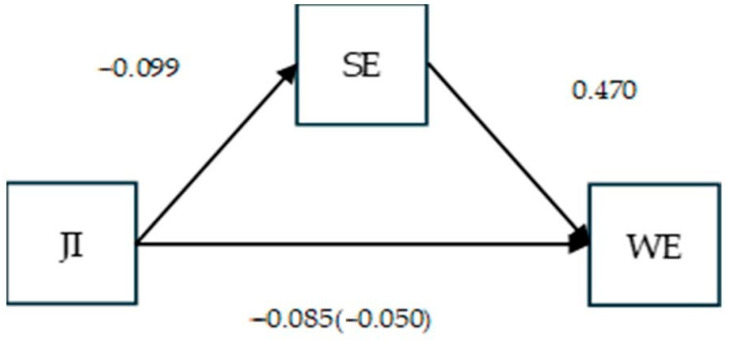
Mediation results for work engagement.

However, the interaction term between self-efficacy and perceived supervisor support was non-significant (B = 0.005; *p* = ns), and thus, the moderated mediation hypothesis, H8, was not supported (Figure 5).

## 5. Discussion

The main purpose of this study was to investigate how job insecurity affects workers’ perceived psychological well-being and work engagement, considering the mediating effect of self-efficacy at work and the moderating effect of perceived supervisor support, thus testing the presence of moderated mediation relationships.

This study shows that job insecurity has a negative impact on work self-efficacy, psychological well-being, and work engagement. Self-efficacy not only positively influences psychological well-being and work engagement but also mediates the effects of job insecurity on these outcomes. Following the COR theory (Hobfoll, 1989), job insecurity threatens essential personal resources, such as self-efficacy; when individuals perceive their jobs as insecure, they experience a loss or threat of resource loss, which undermines their self-efficacy and well-being.

Self-efficacy at work appears to function as a key personal resource with a buffering effect against the resource depletion process. Our finding that the direct effects of job insecurity on psychological well-being and work engagement become smaller through the mediating role of self-efficacy demonstrate its protective role. According to the COR theory, individuals strive to retain, protect, and build resources; thus, self-efficacy can serve as a compensatory mechanism, allowing employees to cope better with the stressor of job insecurity.

Further, we found that higher levels of perceived supervisor support enhance the positive effect of self-efficacy on psychological well-being, which is consistent with the COR’s assertion that social support constitutes a contextual resource that can foster the preservation or restoration of personal resources. However, and somewhat unexpectedly, the negative effects of job insecurity on psychological well-being were also amplified under conditions of high supervisor support. Although this supports the existence of a moderated mediation effect, it does not occur in the hypothesized direction. This may reflect a resource loss spiral, as suggested in the COR theory: when initial losses (e.g., perceived job insecurity) are not effectively buffered, additional resources—such as perceived support—can become strained or ineffective and may even be perceived as inauthentic or threatening, thus compounding the experience of stress.

Regarding work engagement, a similar pattern emerged. Job insecurity diminishes engagement, whereas self-efficacy positively predicts it. Again, the smaller indirect effect of job insecurity via self-efficacy underscores the latter’s role as a personal resource that mitigates the negative consequences of insecurity. While perceived supervisor support has a positive direct effect on both self-efficacy and work engagement, we did not find evidence of its moderating role in the relationship between self-efficacy and engagement. Thus, a full moderated mediation model for engagement could not be supported.

Taken together, these findings underscore the complex interplay of personal and contextual resources in the workplace and offer a nuanced application of the COR theory. While perceived supervisor support is traditionally viewed as a valuable resource that can help employees protect or replenish personal resources (Gist & Mitchell, 1992; Stamper, 2003), our results suggest that under certain conditions, it may fail to fulfill this role or may even exacerbate the experience of resource loss. This aligns with the COR theory’s premise that resource gain is more salient in the context of resource loss, and that the failure of expected resources to materialize or help may intensify stress. As Halbesleben and Buckley (Halbesleben & Buckley, 2004) noted, social support can sometimes backfire, either by masking the underlying stressor, threatening the recipient’s self-esteem, or diminishing over time—each of which can contribute to further resource depletion rather than restoration.

In the Portuguese context, there is another circumstance to consider that may influence our results. Hofstede (1995) identified Portugal’s organizational culture as feminine, collectivist, and characterized by high uncertainty avoidance and power distance. These cultural dimensions significantly influence employees’ perceptions of supervisor support, emphasizing the importance of relationships, group harmony, and collective well-being and thereby making supportive supervisory behavior highly valued.

In organizational cultures that are characterized by high uncertainty avoidance, individuals often feel uncomfortable with ambiguity, leading these organizations to rely heavily on formal rules and hierarchical structures. Although employees may expect emotional support and clear guidance from supervisors, high power distance complicates this dynamic by inhibiting open communication due to an unequal power distribution. Consequently, although supervisor support is desired, employees may hesitate to seek it out of fear of authority (Dai et al., 2022). Additionally, when support is offered, it may be perceived as formal rather than genuine (Durán-Brizuela et al., 2017).

An additional consequence of high power distance is the close scrutiny of supervisors’ behavior. Any perceived change in supervisory support—such as increased attention or feedback—may be interpreted not as a sign of care, but as an indication of underlying issues or potential threats (Dai et al., 2022). In this cultural context, job insecurity may be heightened by limited access to information about organizational changes, in particular when information is mediated by supervisors. In addition, employees’ lack of participation in decision-making and limited empowerment may contribute to feelings of helplessness and diminished control (Dai et al., 2022; Durán-Brizuela et al., 2017).

Besides cultural context, other alternative mechanisms should also be considered. One such explanation concerns the type and quality of the support that is provided by supervisors. In some cases, this support may be perceived as controlling, misaligned with employees’ needs, or undermining their autonomy (Beehr et al., 2010; De Jonge & Dormann, 2007). In insecure work contexts, this may lead to higher perceptions of threat and uncertainty. Additionally, ambiguous or inconsistent support may heighten the psychological strain associated with job insecurity. If supervisors express concern without being able to provide clarity or tangible assistance, this incongruity may deepen the sense of helplessness among employees (Kiewitz et al., 2016; Sverke & Hellgren, 2002).

A further possibility is that this pattern reflects a reverse buffering effect (Lepine et al., 2005), whereby the presence of a typically positive factor (i.e., supervisor support) draws attention to the severity of the negative condition (i.e., job insecurity). The complexity of the supervisor’s role is also exemplified in the work of Yang et al. (2024), who tested the moderation effect of trust in the supervisor in the relationship between psychological well-being and job satisfaction. The relationship was non-significant. According to the authors, their results may have been influenced by national culture or the more contextually contingent nature of trust in the supervisor. It was also argued that different leadership styles, for instance more authoritative or even abusive ones, would have a detrimental effect on well-being by decreasing autonomy and hindering self-development opportunities. In the context of job insecurity, it would also be likely to amplify insecurity perceptions.

The negative effect of job insecurity on work engagement can be explained when considering job insecurity as a stressor and, thus, as producing negative work attitudes and affective responses such as burnout (Bosman et al., 2005; Witte, 1999); with increasing burnout, the levels of work engagement will decrease. When this happens, it is to be expected that the individual resources that are devoted to work will be lower (Saks, 2006) and the positive outcomes of work engagement will be reduced, especially job performance.

The two effects found can be explained as follows (Schaufeli, 2013): assuming that work engagement results from the motivational nature of resources, with job resources being those that either help achieve goals, reduce job demand, or stimulate development, job insecurity will have the opposite effect, thus diminishing work engagement. Personal resources are another source of engagement, associated with resilience, and these comprise self-efficacy, optimism, and emotional stability and have a positive effect on work engagement. In our study, we found that these effects of job insecurity and self-efficacy at work act in opposite directions.

The majority of participants in this study were employed in the private sector, which is typically more exposed to market fluctuations and organizational changes, especially during economic crises (De Witte, 2005). However, most of them held permanent contracts, which are associated with higher job stability and legal protection, thus potentially reducing job insecurity perceptions, even in risk-prone sectors (Cheng & Chan, 2008). The prevalence of permanent contracts may have partially mitigated the impacts of sector-related vulnerabilities on perceived job insecurity.

### 5.1. Theoretical and Practical Implications

This study makes several important contributions both to the discussion of the effects of job insecurity, by using the COR theory as the explanatory framework, and to the COR theory itself.

The tested moderated mediation model showed that job insecurity causes resource depletion by having a negative impact on self-efficacy at work, which was, in turn, shown to be an important resource for buffering job insecurity’s impact on well-being and work engagement.

This study contributes to the COR theory in several ways. Firstly, given the use of a sample collected in the context of the Portuguese debt crisis with a high unemployment rate, it highlights the role of economic instability as an environmental condition that undermines job security (De Witte, 2005). The resulting perceived job insecurity showed a negative impact on psychological well-being, thus reinforcing previous results on the relationship between economic instability and mental health (De Witte et al., 2015).

Secondly, the negative impact of job insecurity on self-efficacy at work provides further evidence of how stress caused by the prospect of job loss weakens this individual resource. At the same time, it highlights its importance as a buffer, since its mediation role diminishes the negative impact of job insecurity on psychological well-being (Hobfoll, 2001). The effects on work engagement follow the same pattern, denoting the same buffering effect of self-efficacy on work engagement.

Thirdly, our results regarding the role of perceived supervisor support, while partially contradicting the hypothesized moderation effect, make a case for the limits of this resource, which can be unhelpful due to organizational culture characteristics or the type or quality of supervision (Beehr et al., 2010; De Jonge & Dormann, 2007; Lepine et al., 2005). Lastly, it also reinforces the importance of recognizing that resources must be viewed in their cultural context. As discussed, perceived supervisor support in a cultural context of high-power distance may act differently than in other cultures; in organizations with a lower power distance, the results will probably be different, and support from a supervisor may be a source of help and a valuable resource.

Considering the continuous and rapid evolution of technology and markets, urging companies to adapt to their ever-changing environments, workforces are likely to be more affected by threats to their jobs, either due to tasks being eliminated or industries turning obsolete; to this, we can add the growing employment of non-permanent workers to allow for swift adjustments in workforces, leading to growing perceptions of job insecurity among workers. This leads to decreased well-being and work engagement, potentially negatively impacting productivity and organizational efficiency and leading to poorer bottom-line results. Promoting self-efficacy in the workforce appears to be a way to at least partially counteract these undesirable outcomes; it can also be a way to increase individual resources to help with stress responses.

Organizations can introduce various strategies to increase or maintain employees’ self-efficacy to counteract the effects of job insecurity. Bandura (1997) suggests that mastery experiences, i.e., completing tasks successfully, would be a way to increase self-efficacy. Another strategy would be to redesign work, allowing individuals to reach progressive achievements and giving them timely feedback, which can also support this process. In the work by F. Wang and Ding (2023), we find the suggestion of strengths-based leadership, which entails managers helping employees to recognize and use their unique talents, as a way of increasing self-efficacy (F. Wang & Ding, 2023).

Other possible avenues would be observational learning, since witnessing peers managing job insecurity can further strengthen self-efficacy (Luthans & Youssef, 2007); alternatively, Psychological Capital (PsyCap) development programs, which include self-efficacy, hope, resilience, and optimism, could also be used to mitigate the negative effects of job insecurity (Avey et al., 2009). Training and development programs are also a way to promote adaptability and consequently reduce perceptions of job insecurity (F. Wang & Ding, 2023; Yao et al., 2021).

### 5.2. Limitations and Suggestions for Future Work

There are some limitations to our study. The primary constraint is associated with the cross-sectional design of this study, which does not allow us to assume causal relationships between the variables.

The utilization of single-item measures for job insecurity and self-efficacy at work in this study may be considered a potential limitation. While multi-item measures are generally preferred for addressing complex constructs and enhancing measurement precision, single-item measures offer certain advantages, including the facilitation of more concise surveys and reduced criterion contamination (Podsakoff et al., 2003). It is worth noting that a study examining the validity of single-item measures (Matthews et al., 2022) demonstrated that individual items assessing job insecurity and job self-efficacy exhibited very good validity. This finding lends support to the methodological approach employed in the present investigation.

Another limitation of this study is that the hypotheses were tested using only self-reported measures, which may introduce common method bias. However, we conducted a Harman single-factor test to mitigate this concern, as well as a marker variable test, and the results of both tests indicated that common method bias is unlikely to have influenced the results presented in this study.

In our view, future research should continue to analyze job insecurity’s relationships with different outcomes at the individual and organizational levels.

For the study of relationships between job insecurity, psychological well-being, and work engagement, other mediators and moderators can be used, such as analyzing the role of coping strategies in moderating individual responses to the stress associated with job insecurity, as exemplified by Lopez Bohle et al. (2024), who showed that the different effects of various coping strategies mediated the relationship between job insecurity and a range of outcomes. Psychological contract breach, based on social exchange theory, can be another potential mediator, as in the work by Costa and Neves (2017), it was found that psychological contract breach mediated the relationship between job insecurity and in-role performance and organizational deviance. Moreover, perceptions of organizational justice have also been studied as a moderator in the job insecurity literature, with evidence suggesting that high perceptions of organizational justice can mitigate the negative effects of job insecurity on outcomes such as job performance, burnout, and work engagement (De Angelis et al., 2021; H. Wang et al., 2015).

To enhance the discussion, future studies could benefit from a comparative evaluation of various countries and organizational cultures, specifically those with varying scores on cultural dimensions such as power distance and collectivism. Analyzing cultures with high power distances in contrast to ones with low ones may uncover significant distinctions in the experience of perceived supervisor support. In addition, comparisons between collectivist cultures and more individualist ones could clarify different expectations of supervisor support. Cross-cultural studies could provide a more comprehensive understanding and contribute to a more refined interpretation of the relationship between national and organizational cultures and perceived supervisor support.

Researchers should also make use of longitudinal studies, not only to establish causality (Taris & Kompier, 2003) but also to unveil changes over time under prolonged job insecurity. For example, measuring job insecurity first and then, in sequence, its effects on self-efficacy or supervisor support perceptions, would clarify a possible effect of prolonged job insecurity perceptions on the erosion of self-efficacy (König et al., 2010) and/or whether the same erosion extends to support our understanding of these issues. A longitudinal design would also clarify how low levels of support could aggravate insecurity’s impact over time, or how self-efficacy mitigates resource depletion and to what extent. Seizing resource dynamics is crucial, since the COR theory emphasizes resource loss spirals and gain cycles.

Longitudinal research can also be useful for probing non-linear relationships. An example is the study by Selenko et al. (2013), which presented evidence of a non-linear relationship between job insecurity and self-rated job performance. At lower and moderate levels of job insecurity, the effect on performance was more negative than at higher levels of job insecurity. Job insecurity also predicted performance over a one-year period. Furthermore, the study evidenced that optimism had a positive effect on performance, which also remained over one year, unlike supervisory support, whose effect was only felt at the first moment of data collection and not after a year (Selenko et al., 2013).

## 6. Conclusions

The 2015 financial crisis in Portugal, which affected the nation’s economy and labor market, may have exerted an influence on our study outcomes. It is important to note that economic conditions and unemployment rates are antecedents of perceived job insecurity. Recent data from Banco de Portugal indicates that in the first quarter of 2025, the overall unemployment rate was 6.4%. Although this figure represents a clear improvement from the 2015 level of 12.5%, it remains substantial, and perceived job insecurity is a reality for many workers.

Our study responds to the need for more research on threats to workers’ well-being and work engagement, such as job insecurity, while exploring possible safeguards. Specifically, we developed and tested a model examining self-efficacy as a mediating mechanism and supervisor support as a moderator of job insecurity’s relationships with well-being and work engagement. In our study, we presented a moderated mediation model that enables a more nuanced understanding of how job insecurity influences perceived well-being and work engagement when in the presence of self-efficacy and at different levels of supervisor support. We found that self-efficacy attenuates the negative impact of job insecurity on well-being and engagement, but in the case of well-being, higher levels of support from a supervisor have a more damaging effect, which may be due to the contextual effect of the organizational culture in Portugal or, as pointed out earlier, to a depletion of that support over time or even threats to self-esteem. We encourage future research to address this particular subject to offer more insights.

## Figures and Tables

**Table 1:** Descriptive statistics, Cronbach’s alpha, and correlation coefficients of variables.

Variable	Mean	SD	α	1	2	3	4	5	6	7	8	9
1. Age	41.4	13	-	-	-	-	-	-	-	-	-	-
2. Gender	0.43	-	-	0.013	-	-	-	-	-	-	-	-
3. Tenure	14.5	22.3	-	0.286 **	0.058	-	-	-	-	-	-	-
4. Contract	0.63	-	-	0.093 **	−0.016	0.098 **	-	-	-	-	-	-
5. Job insecurity	2.58	1.12	-	0.001	0.016	−0.042	−0.156 **	-	-	-	-	-
6. Self-efficacy	4.23	0.75	-	−0.035	−0.063 *	−0.030	0.123 **	−0.144 **	-	-	-	-
7. Supervisor support	3.82	0.83	0.92	−0.059	−0.054	−0.038	0.128 **	−0.220 **	0.195 **	-	-	-
8. Work engagement	3.89	0.73	0.78	−0.076 *	−0.069 *	−0.045	0.129 **	−0.208 **	0.560 **	0.464 **	-	-
9. Well-being	3.36	0.89	0.90	−0.138 **	−0.013	−0.019	0.093 **	−0.240 **	0.302 **	0.424 **	0.532 **	-

Note: N = 938; SD = standard deviation; α = Cronbach’s alpha; * *p* < 0.05, ** *p* < 0.01. Sex: dummy-coded as 1 = male. Contract: dummy-coded as 1 = permanent.

**Table 2:** Hierarchical regression analyses.

Variable	Well-Being				Work Engagement			
	Model 1	Model 2	Model 3	Model 4	Model 1	Model 2	Model 3	Model 4
1. Age	−0.104 *	−0.099 *	−0.095 *	−0.067 *	−0.056	−0.051	−0.043	−0.025
2. Gender	0.016	0.014	0.037	0.041	−0.051	−0.053	−0.009	−0.014
3. Tenure	0.033	0.023	0.025	0.038	−0.005	−0.014	−0.01	−0.015
4. Contract	0.121 ***	0.082 *	0.045	0.025	0.138 ***	0.104 ***	0.033	0.022
5. Job insecurity		−0.230 ***	−0.190 ***	−0.130 ***		−0.202 ***	−0.125 ***	−0.062 **
6. Self-efficacy			0.282 ***	0.239 ***			0.540 ***	0.491 ***
7. Supervisor Support				0.335 ***				0.347 ***
R^2^	0.025	0.076	0.156	0.260	0.028	0.065	0.346	0.457
Δ R^2^		0.051	0.080	0.104		0.038	0.280	0.111
F	4.79 ***	38.35 ***	65.07 ***	96.17 ***	4.87 ***	27.95 ***	295.01 ***	140.24 ***

Note: N = 983; * *p* < 0.05, ** *p* < 0.01, *** *p* < 0.001.

**Table 3:** Effects of job insecurity, self-efficacy, and supervisor support on psychological well-being—direct, indirect, and conditional effects.

	Consequent						
	Self-Efficacy			Well-Being			
Antecedent	Coeff.	SE	*p*	Coeff.	SE		*p*
Job insecurity (JI)	−0.099	0.021	<0.001	−0.106	0.023		<0.001
Self-efficacy (SE)	-	-	-	0.290	0.036		<0.001
Supervisor support (SS)	-	-	-	0.355	0.033		<0.001
SE × SS	-	-	-	0.120	0.039		<0.001
R^2^ = 0.023				R^2^ = 0.256			
F(1.872) = 20.610, *p* < 0.001				F(4.869) = 74.964, *p* < 0.001			
Total, direct, and indirect effects of job insecurity on well-being							
				Effect	SE		*p*
Total effect				−0.193	0.024		<0.001
Direct effect				−0.161	0.024		<0.001
				Indirect effect	Boot SE	Boot LLCI	Boot ULCI
Indirect effect (through self-efficacy)				−0.037 (0.039)	0.009	−0.059	−0.021
Conditional direct effects of self-efficacy on well-being							
Moderator: Supervisor support				Effect		*p*	
−1 SD				0.19		<0.001	
Mean				0.29		<0.001	
+1 SD				0.39		<0.005	
Conditional indirect effects of job insecurity on well-being							
Moderator: Supervisor support				Effect	LLCI		ULCI
−1 SD				−0.018	−0.033		−0.008
Mean				−0.028	−0.043		−0.015
+1 SD				−0.038	−0.058		−0.019

Note: standardized coefficients are placed in brackets.

**Table 4:** Effects of job insecurity, self-efficacy, and supervisor support on work engagement—total, direct, and indirect effects.

	Consequent						
	Self-Efficacy			Work Engagement			
Antecedent	Coeff.	SE	*p*	Coeff.	SE		*p*
Job insecurity (JI)	−0.099	0.021	<0.001	−0.040	0.016		<0.010
Self-efficacy (SE)	-	-	-	0.47	0.025		<0.001
Supervisor support (SS)	-	-	-	0.317	0.023		<0.001
SE × SS	-	-	-	0.005	0.027		ns
R^2^ = 0.023				R^2^ = 0.453			
F(1.872) = 20.610, *p* < 0.001				F(4.869) = 180.115, *p* < 0.001			
Total, direct, and indirect effects of job insecurity on work engagement							
				Effect	SE		*p*
Total effect				−0.136	0.022		<0.001
Direct effect				−0.085	0.017		<0.001
				Indirect effect	Boot SE	Boot LLCI	Boot ULCI
Indirect effect (through self-efficacy)				−0.050 (0.077)	0.018	−0.113	−0.041

Note: standardized coefficients are placed in brackets.

## Data Availability

The raw data supporting the conclusions of this article is available at https://www.eurofound.europa.eu/en/surveys. The data that are used in the current study are secondary data. Data source: https://www.eurofound.europa.eu/en/surveys. Data availability: https://www.eurofound.europa.eu/en/surveys/about-eurofounds-surveys/data-availability, accessed on 20 April 2021. The data are available free of charge to those who intend to use them for non-commercial purposes.
